# Identifying and characterising Thrap3, Bclaf1 and Erh interactions using cross-linking mass spectrometry

**DOI:** 10.12688/wellcomeopenres.17160.2

**Published:** 2023-01-06

**Authors:** Liudmila Shcherbakova, Mercedes Pardo, Theodoros Roumeliotis, Jyoti Choudhary

**Affiliations:** 1 Cancer Biology, Institute of Cancer Research, UK, London, UK

**Keywords:** Endogenous complexes, XL-MS, CLMS cross-linking mass spectrometry, DSSO, Thrap3, Bclaf1, Erh

## Abstract

**Background: **Cross-linking mass spectrometry (XL-MS) is a powerful technology capable of yielding structural insights across the complex cellular protein interaction network. However, up to date most of the studies utilising XL-MS to characterise individual protein complexes’ topology have been carried out on over-expressed or recombinant proteins, which might not accurately represent native cellular conditions.

**Methods:** We performed XL-MS using MS-cleavable crosslinker disuccinimidyl sulfoxide (DSSO) after immunoprecipitation of endogenous BRG/Brahma-associated factors (BAF) complex and co-purifying proteins. Data are available via ProteomeXchange with identifier PXD027611.

**Results:** Although we did not detect the expected enrichment of crosslinks within the BAF complex, we identified numerous crosslinks between three co-purifying proteins, namely Thrap3, Bclaf1 and Erh. Thrap3 and Bclaf1 are mostly disordered proteins for which no 3D structure is available. The XL data allowed us to map interaction surfaces on these proteins, which overlap with the non-disordered portions of both proteins. The identified XLs are in agreement with homology-modelled structures suggesting that the interaction surfaces are globular.

**Conclusions:** Our data shows that MS-cleavable crosslinker DSSO can be used to characterise in detail the topology and interaction surfaces of endogenous protein complexes without the need for overexpression. We demonstrate that Bclaf1, Erh and Thrap3 interact closely with each other, suggesting they might form a novel complex, hereby referred to as TEB complex. This data can be exploited for modelling protein-protein docking to characterise the three-dimensional structure of the complex. Endogenous XL-MS might be challenging due to crosslinker accessibility, protein complex abundance or isolation efficiency, and require further optimisation for some complexes like the BAF complex to detect a substantial number of crosslinks.

## Introduction

Cross-linking mass spectrometry (XL-MS) is a powerful technique that enables the identification of proximal amino acid residues within a single protein as well as residues in close proximity in interacting proteins. Intramolecular crosslinks provide distance constraint parameters that can support homology-based tertiary structure modelling or even guide *de novo* modelling ( Adikaram *et al.*, 2019; Kahraman *et al.*, 2013; Liu *et al.*, 2020; Orbán-Németh *et al.*, 2018; O'Reilly & Rappsilber, 2018). Moreover, intermolecular cross-linking information has been used to determine spatial orientation and elucidate the topology of protein complex subunits ( Gaik *et al.*, 2015; Herzog *et al.*, 2012; Leitner *et al.*, 2016; Politis *et al.*, 2014). In the absence of a full tertiary structure model protein-protein interaction (PPI) surface information derived from XL-MS can potentially serve as a guide to direct mutation or small molecule screens to disrupt specific subunit interactions within a given complex.

Most XL-MS studies to date have been performed on over-expressed or recombinant proteins or protein complexes ( O'Reilly & Rappsilber, 2018). More rarely, XL-MS has been applied to study protein interactions in a more complex endogenous setting like cell lysates ( Götze *et al.*, 2019; Klykov *et al.*, 2018; Liu *et al.*, 2015; Yugandhar *et al.*, 2020), organelles ( Bartolec *et al.*, 2020; Makepeace *et al.*, 2020; Ryl *et al.*, 2020) specific cellular compartments ( Fasci *et al.*, 2018; Ser *et al.*, 2019) or purified complexes expressed at endogenous levels ( Makowski *et al.*, 2016; Spruijt *et al.*, 2021). Recent advances in XL-MS have introduced MS-cleavable crosslinkers, which generate distinct fragment pairs in MS2 and therefore substantially reduce the complexity of data analysis and improve identification accuracy ( Matzinger & Mechtler, 2021). Disuccinimidyl sulfoxide (DSSO) ( Kao *et al.*, 2011) is the most extensively used MS-cleavable reagent, and has been applied to the characterisation of protein interactions both in isolated proteins as well as complex samples ( Adams *et al.*, 2020; Klykov *et al.*, 2018; Nguyen *et al.*, 2020; Ser *et al.*, 2019; Smith *et al.*, 2018; Wang *et al.*, 2017).

Here, we performed XL-MS on affinity-purified (AP) endogenous BRG/Brahma-associated factors (BAF) complex in native conditions to define the interaction surfaces between complex subunits and with associated protein partners. The strategy was based on immunoprecipitation of Arid1a, considered to be a scaffolding/bridging component of the complex ( Han *et al.*, 2020; He *et al.*, 2020; Mashtalir *et al.*, 2018), and unlike most published XL-MS studies, involved no artificial increase of the target protein complex by overexpression. In addition to BAF purification, we concomitantly achieved a significant co-enrichment of Thrap3, Bclaf1 and Erh proteins that allowed the identification of a substantial number of crosslinks (XLs) between them, implicating this protein cluster as a native protein assembly, that we hereby refer to as TEB complex. We report the interaction surfaces between Thrap3, Bclaf1 and Erh, determined through chemical crosslinking mass-spectrometry.

## Results

### Bclaf1, Erh and Thrap3 interact directly with each other

To investigate interaction surfaces of Arid1a and the BAF complex, we carried out five experiments where immunoprecipitation of endogenous Arid1a from mouse embryonic stem cells (mESCs) was coupled to crosslinking using MS cleavable crosslinker DSSO and MS3 mass spectrometry. Crosslink peptide identification was performed using the full mouse Uniprot database, with crosslink assignment using XlinkX ( Liu *et al.*, 2015) as a part of Proteome Discoverer at a 1% false discovery rate (FDR).

Whilst we were able to detect several XLs between BAF subunits and interacting proteins, these were mostly single crosslink spectra matches (CSMs) with exception of two. We also detected a significant number of XLs between Thrap3, Erh and Bclaf1, due to a strong enrichment of these proteins in Arid1a APs. Specifically, 13% of all XLs identified in the five experiments involved these three proteins. For comparison, 9.6% of total XLs obtained were assigned to BAF, which, in addition to being the bait in the AP, is also a much larger complex with 27 subunits. Given the richness of XL information associated with these proteins, we used this data to gain insight into the structural topology of this protein cluster. Our data suggests that they interact closely and might represent a stable complex. We hereby refer to it as the Bclaf1-Erh-Thrap3 (TEB) complex.

**Figure 1.**
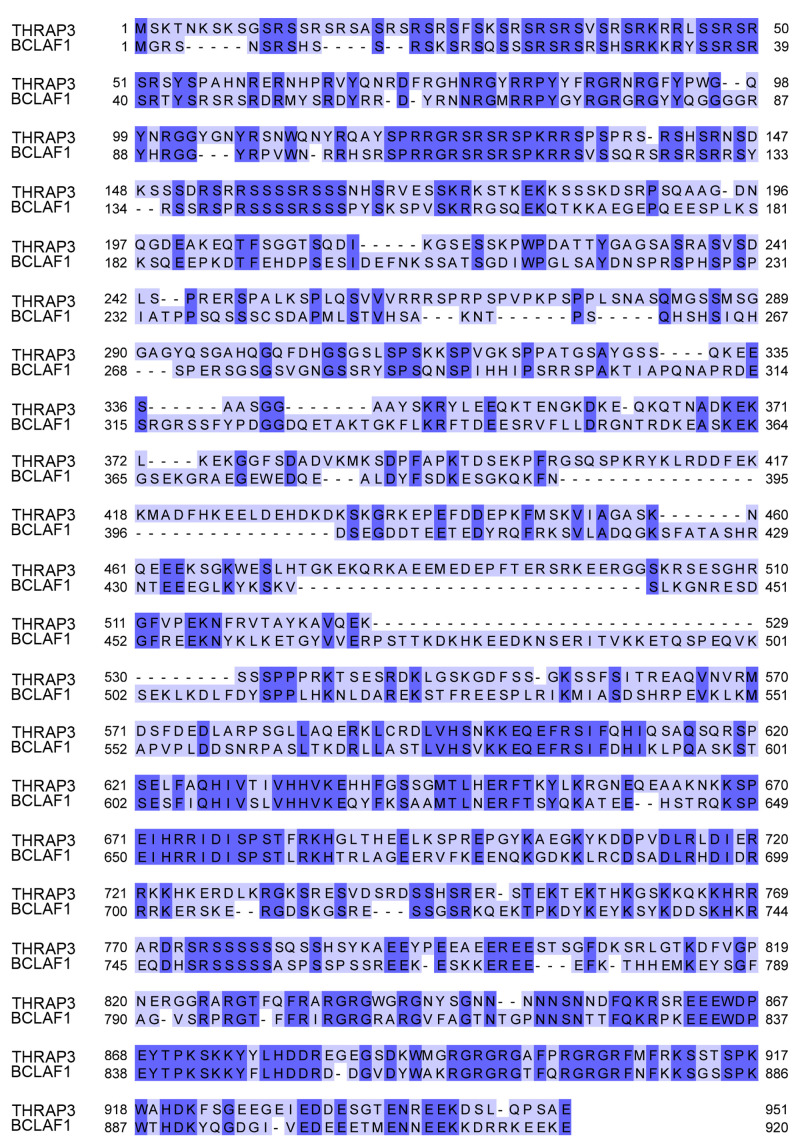
Protein sequence alignment of mouse Thrap3 and mouse Bclaf1. Proteins showed ~43% homology. The alignment was performed using Clustal Omega sequence alignment tool ( Madeira *et al.*, 2019) within the Jalview v2.11.1.4 ( Waterhouse *et al.,* 2009).

**Figure 2.**
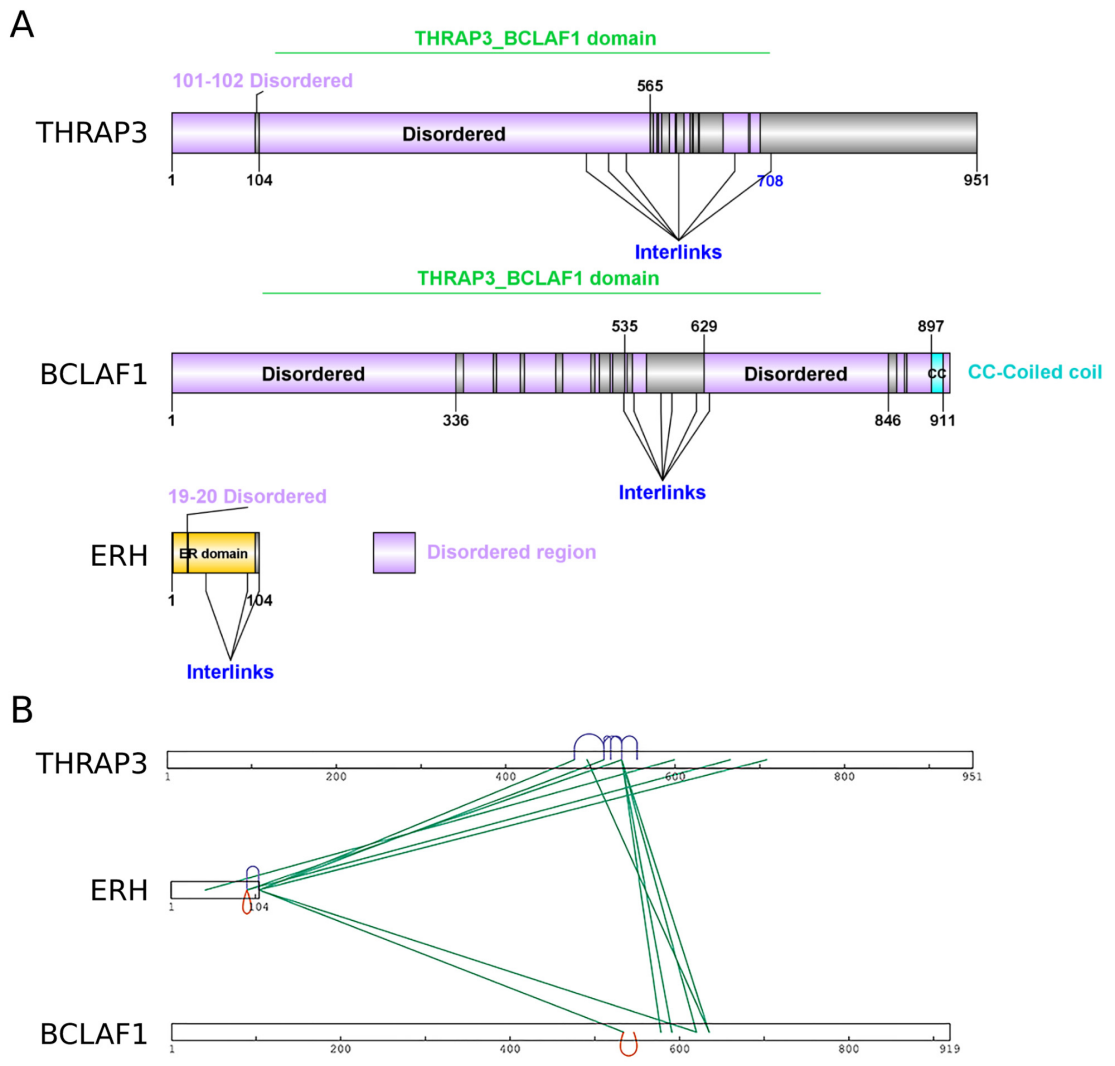
Structural features of TEB complex members and identified cross-linked sites. ( **A**) Annotations of domains and disorder regions of Thrap3, Bclaf1 and Erh as reported in Pfam database ( Mistry *et al.*, 2021). The illustration was made using DOG 2.0 ( Ren *et al.*, 2009). ( **B**) A two dimensional visualisation of Thrap3, Bclaf1 and Erh depicting the high confidence cross-links using xiVIEW ( Graham *et al.*, 2019).

Bclaf1 and Thrap3 are highly homologous proteins ( Figure 1). They share the Thrap3-Bclaf1 domain, which defines the THRAP3/BCLAF1 family, that contains these two proteins. There is no available crystal or cryo-electron microscopy structure for Thrap3 or Bclaf1. Interestingly, domain analysis with Pfam database (v33.1) ( Mistry *et al*., 2021) showed that Thrap3 and Bclaf1 are both highly disordered proteins along the whole length ( Figure 2A), which limits the application of structure prediction modelling on them without additional information. Hence our XL-MS data could add useful information for structural elucidation of the complex.

We summarised confident crosslinks and corresponding number of CSMs for each of five experiments in Table 1. Due to the high sequence homology between Thrap3 and Bclaf1 ( Figure 1), the crosslinks were manually checked for unambiguous assignment between the two proteins and undistinguishable pairs were removed from analysis. We detected 24 unique XL sites and a total of 121 CSMs across all five experiments. The high density of crosslinks in the regions from 496-537 amino acids (aa) for Thrap3 and 578-635aa for Bclaf1 ( Figure 2B) indicates the interaction surface between the two proteins. This region of Thrap3 also contains most of XLs to-self, suggesting that it is a potentially globular area and is used for PPI. On the contrary, no intra-links were retained for Bclaf1 upon high confidence filtering ( Figure 2B). Even though they share ~43% homology, the middle parts of these proteins are quite different between approximately 400aa and 560aa, with fragments missing in one protein or the other ( Figure 1). The Bclaf1 and Thrap3 interaction interface also contains their cross-linked sites to Erh, which extends slightly further (up to 481-708aa in Thrap3 and 534-620aa in Bclaf1). Moreover, the region of dense crosslinks in Bclaf1 lies outside of the disordered region as reported in Pfam database ( Mistry *et al*., 2021) (561-629aa; Figure 2A), suggesting that the Bclaf1 interaction surface, like that of Thrap3, may also be a globular structure. Our data demonstrates that Bclaf1, Erh and Thrap3 interaction is direct and suggests that they might form a ternary complex which we have named TEB (Thrap3-Erh-Bclaf1a).

**Table 1.** TEB complex confident crosslinks. This table reports the confident crosslinks, which are defined as being detected in more than one experiment, and its corresponding CSMs number for each of five experiments. XL-pairs ID indicate peptide that contains crosslink to the unique site pair. For each unique crosslinked peptide pair we report the crosslinked spectra match (CSMs) count for each individual experiment. All CSMs fulfill the 1% FDR cut-off.

XL- pair_ID	Crosslink Type	Sequence A	Accession A	Protein Descriptions A	Position A	Sequence B	Accession B	Protein Descriptions B	Position B	Exp.1 #CSMs	Exp.2 #CSMs	Exp.3 #CSMs	Exp.4 #CSMs	Exp.5 #CSMs
1	Inter	EESTSGFD[K]SR	Q569Z6	Thrap3	808	LLIY[K]VSNR	P01631	Ig kappa chain V-II region 26–10	55	1	0	4	5	0
2	Inter	Y[K]DDPVDLR	Q569Z6	Thrap3	708	QAQQAG[K]	P84089	Erh	104	0	0	1	1	0
3	Inter	GNEQEAA[K]NKK	Q569Z6	Thrap3	665	E[K]IYVLLR	P84089	Erh	90	0	0	1	1	0
4	Inter	FTSYQ[K]ATEEHSTR	Q8K019	Bclaf1	635	SR[K]EER	Q569Z6	Thrap3	496	2	0	3	0	0
5	Inter	FTSYQ[K]ATEEHSTR	Q8K019	Bclaf1	635	[K]TSESR	Q569Z6	Thrap3	537	1	0	2	0	1
6	Inter	EQYF[K]SPAVTLNER	Q8K019	Bclaf1	620	QAQQAG[K]	P84089	Erh	104	0	1	0	1	3
7	Inter	EQYF[K]SPAVTLNER	Q8K019	Bclaf1	620	[K]TSESR	Q569Z6	Thrap3	537	1	0	2	0	3
8	Inter	SIFDHI[K]LPQANK	Q8K019	Bclaf1	591	[K]TSESR	Q569Z6	Thrap3	537	0	0	1	1	0
9	Inter	LLASTLVHSV[K]K	Q8K019	Bclaf1	578	[K]TSESR	Q569Z6	Thrap3	537	0	0	1	1	0
10	Intra	GDFSSG[K]SSFSITR	Q569Z6	Thrap3	555	[K]TSESR	Q569Z6	Thrap3	537	0	0	4	1	0
11	Inter	I[K]MIASDSHRPEVK	Q8K019	Bclaf1	534	QAQQAG[K]	P84089	Erh	104	1	0	1	0	0
12	Intra	I[K]MIASDSHRPEVK	Q8K019	Bclaf1	534	MIASDSHRPEV[K]LK	Q8K019	Bclaf1a	546	0	0	0	0	6
13	Intra	VTAY[K]AVQEK	Q569Z6	Thrap3	524	GFVPE[K]NFR	Q569Z6	Thrap3	516	0	0	1	1	0
14	Intra	VTAY[K]AVQEK	Q569Z6	Thrap3	524	[K]TSESR	Q569Z6	Thrap3	537	1	0	4	0	0
15	Inter	GFVPE[K]NFR	Q569Z6	Thrap3	516	RQAQQAG[K]	P84089	Erh	104	2	0	4	0	3
	Inter	GFVPE[K]NFR	Q569Z6	Thrap3	516	QAQQAG[K]	P84089	Erh	104	0	0	0	5	0
16	Intra	GFVPE[K]NFR	Q569Z6	Thrap3	516	[K]TSESR	Q569Z6	Thrap3	537	0	0	1	2	0
17	Inter	[K]AEEMEDEPFTER	Q569Z6	Thrap3	481	QAQQAG[K]	P84089	Erh	104	0	1	1	1	0
	Inter	[K]AEEMEDEPFTER	Q569Z6	Thrap3	481	RQAQQAG[K]	P84089	Erh	104	0	0	0	0	3
18	Intra	[K]AEEMEDEPFTER	Q569Z6	Thrap3	481	GFVPE[K]NFR	Q569Z6	Thrap3	516	0	0	0	0	3
19	Intra	E[K]IYVLLRR	P84089	Erh	90	E[K]IYVLLR	P84089	Erh	90	0	0	1	2	5
20	Intra	E[K]IYVLLR	P84089	Erh	90	RQAQQAG[K]	P84089	Erh	104	0	0	2	2	1
21	Inter	E[K]IYVLLR	P84089	Erh	90	[K]TSESR	Q569Z6	Thrap3	537	0	0	4	3	1
22	Inter	FSGSGSGTDFTL[K]ISR	P01631	Ig kappa chain V-II region 26–10	79	EESTSGFD[K]SR	Q569Z6	Thrap3	808	0	0	3	4	4
23	Inter	FSGSGSGTDFTL[K]ISR	P01631	Ig kappa chain V-II region 26–10	79	EE[K]DSLQPSAE	Q569Z6	Thrap3	943	0	0	1	1	1
24	Inter	MYEEHL[K]R	P84089	Erh	41	DLVHSN[K]K	Q569Z6	Thrap3	599	1	0	1	0	0

Previous studies identified that both the N-terminus (1-190aa) and the C-terminus (359-951aa) of Thrap3 are important for its function in DNA repair of post double stranded breaks and stalled replication forks, whilst the residues 190-359aa appear to be dispensable for this function ( Vohhodina *et al.*, 2017). The C-terminal fragment of Thrap3 has been shown to partially rescue the Thrap3 knockout phenotype in terms of ability to respond to ionising radiation (IR) induced DNA damage ( Vohhodina *et al.*, 2017). Since this Thrap3 C-terminal fragment covers the XL-rich region, the rescue may have been mediated by restoration of interactions with Erh and Bclaf1. Moreover, there is a number of highly frequent phosphorylation sites (>100 reports per site) reported in PhosphoSitePlus (v6.6.0.1) ( Hornbeck *et al.*, 2015) within the Thrap3 C-terminus in mice (S379, S572, S679, S924).

### Mapping TEB crosslinks on available structures

**Figure 3.**
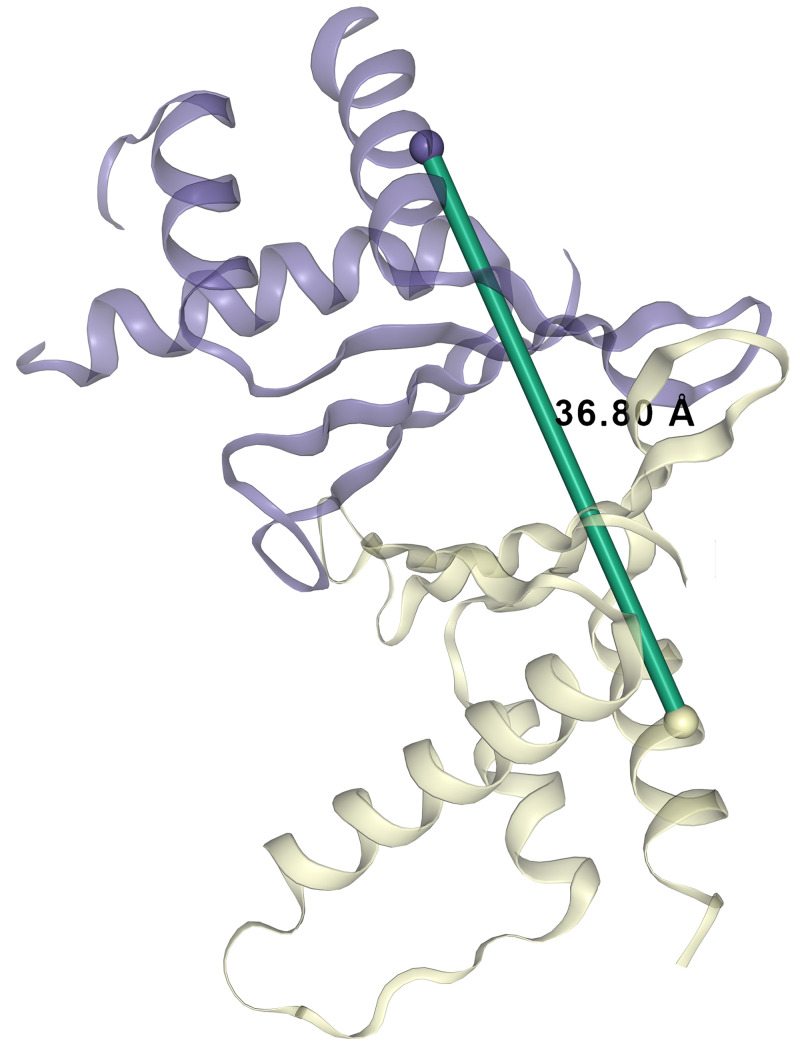
Erh dimer inter-crosslink. ERH inter-crosslink at position K90-K90 was mapped onto available Erh (Uniprot accession: P84089) structure PDB:1WZ7 ( Arai *et al.*, 2005).

Erh is the only component of the TEB complex with an available crystal structure ( Arai *et al.*, 2005; Hazra *et al.*, 2020; Jin *et al.*, 2007; Kwon *et al.*, 2020; Li *et al.*, 2005; Wan *et al.*, 2005; Xie *et al.*, 2019). We detected a XL for Erh that involves two connected peptides with overlapping sequences ( Table 1; XL-pair ID 19). Although XL-MS is not able to distinguish between intra- or inter-links in the case of homo-multimeric proteins, the overlap in the crosslinked sequences conclusively identifies an inter-molecular interaction, that is, a crosslink involving two molecules of the same protein (or alternatively a false positive peptide identification). In agreement with this data, Erh is known to form a homodimer ( Arai *et al.*, 2005; Hazra *et al.*, 2020; Xie *et al.*, 2019) and therefore we concluded that the self-link at the position 90 involves two Erh molecules. We utilised available Erh homodimer protein structure PDB:1WZ7 ( Arai *et al.*, 2005) to model this crosslink within a structural context ( Figure 3). The length of the mapped crosslink is in agreement with the DSSO maximum distance constraint of 37Å threshold used in other modelling studies ( Liu *et al.*, 2020).

### TEB crosslinks satisfy the model based on sequence homology

**Figure 4.**
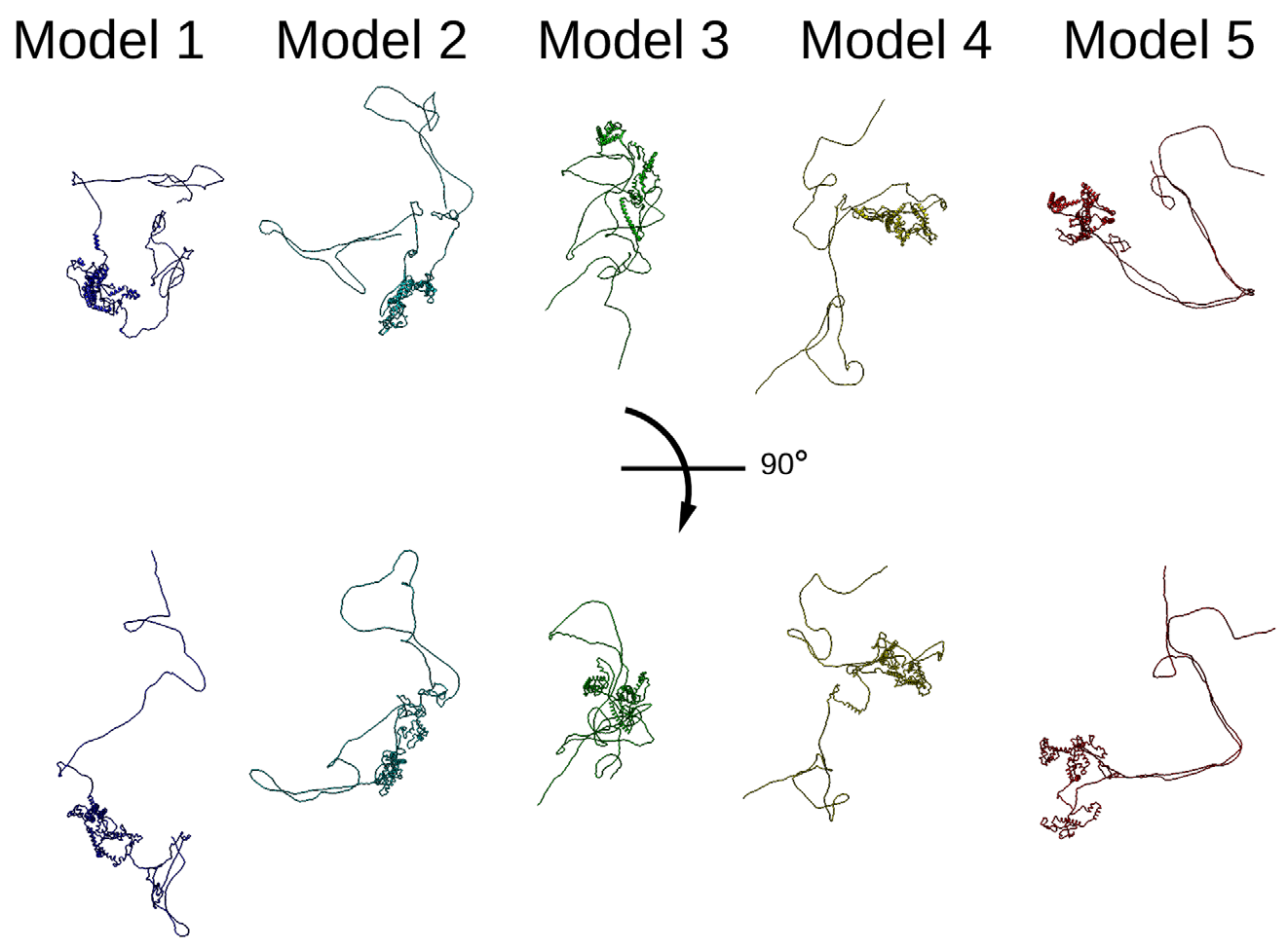
Thrap3 sequence-based modelling. Thrap3 (mouse) protein models generated using Robetta protein modelling tool ( Raman *et al.*, 2009; Song *et al.*, 2013) based solely on the protein sequence.

**Figure 5.**
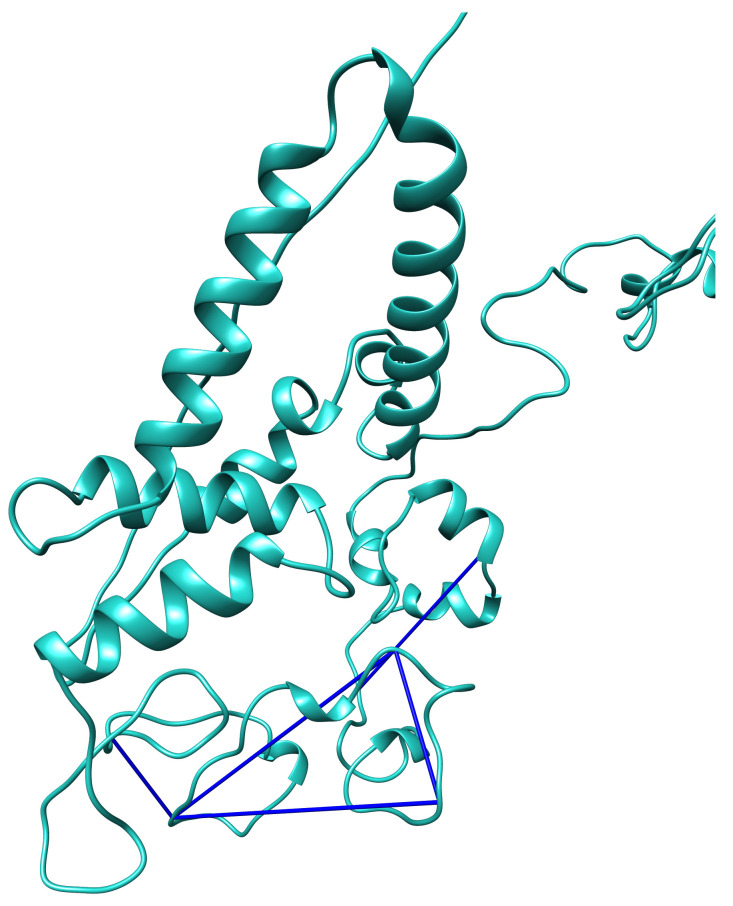
Thrap3 crosslinks satisfy the *de novo* homology-based model. Predicted zoomed in structure of Thrap3 model 2 using Robetta protein modelling tool ( Raman *et al.*, 2009; Song *et al.*, 2013). Cross-linking data was mapped using Chimera with distance constraint set at 32Å ( Pettersen *et al.*, 2004).

Since there is no available crystal or cryo-EM structure for Thrap3 or Bclaf1, we used online modelling tool Robetta for *de novo* modelling of putative structures ( Raman *et al.*, 2009; Song *et al.*, 2013), and five predicted structures were rendered for each protein. In all models Thrap3 appeared to be largely unfolded and flexible ( Figure 4), with roughly the same area stretching from 330-725aa folding into a number of helices that resemble a globular structure. This region overlapped with the crosslink-dense area of Thrap3 ( Figure 5). When XLs were mapped onto the proposed models 5 out of the 5 crosslinks we detected satisfied the more restrictive distance threshold of 32Å ( Armony *et al.*, 2021) in models 2, 3 and 5, whilst 4 out 5 did in models 1 and 4.

**Figure 6.**
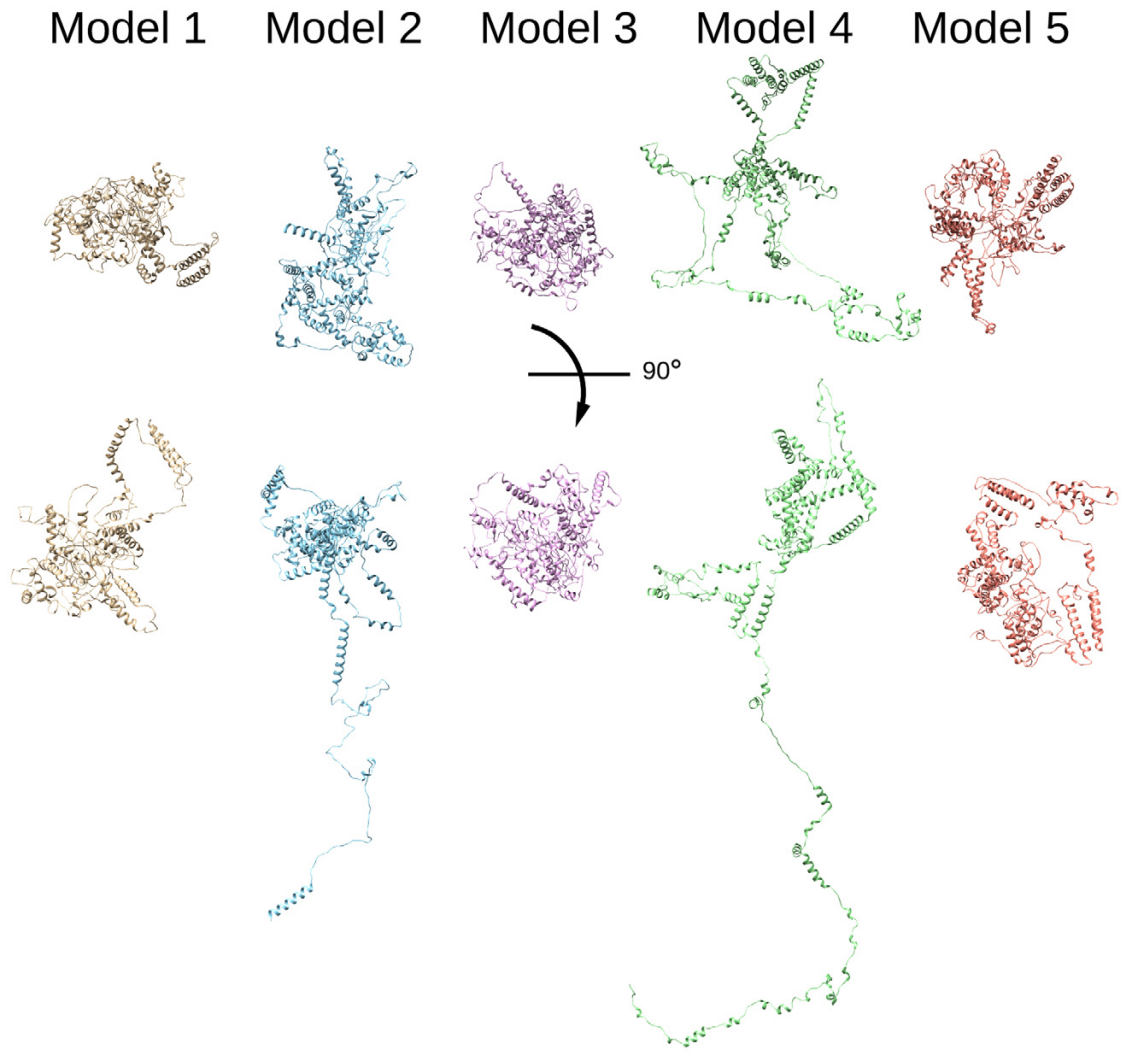
Bclaf1 sequence-based modelling. Bclaf1 (mouse) protein model generated using Robetta protein modelling tool ( Raman *et al.*, 2009; Song *et al.*, 2013) based solely on the protein sequence.

Surprisingly, despite the homology between Bclaf1 and Thrap3, the predicted structures for Bclaf1 appeared generally much more folded ( Figure 6) than those of Thrap3 and overall looked very different.

## Discussion

Thrap3 and Bclaf1 have been shown to form part of a high molecular weight complex (the SNARP complex) involved in the regulation of cyclin D1 stability together with SNIP1, SkIP and Pinin ( Bracken *et al.*, 2008). Interactions between Bclaf1 and Thrap3 with Erh have been identified through immunoprecipitation of Erh ( Kavanaugh *et al.*, 2015), but these interactions were not further validated. Our XL-MS data confirms that Bclaf1, Thrap3 and Erh are in close proximity to each other and provides evidence that they can interact directly. Furthermore, the experimental conditions of cell lysis (high salt buffer) and the number of XLs detected suggest that they form a tight complex that we refer to as the TEB complex. Bclaf1, Erh and Thrap3 are functionally related and are all involved in splicing regulation. Thrap3 and Bclaf1 have also been shown to play an important role in DNA damage repair (DDR) through regulation of splicing of ataxia telangiectasia mutated *(ATM)* serine/threonine kinase and export of its mRNA, as well as regulation of other transcripts involved in DDR ( Vohhodina *et al.*, 2017), while Erh has been reported to regulate splicing of ataxia telangiectasia and Rad3-related ( *ATR*) protein ( Kavanaugh *et al.*, 2015). Thrap3 and Bclaf1 are able to compensate for the loss of each other ( Vohhodina *et al.*, 2017) indicating partial functional redundancy.

There is a discrepancy in the literature as to whether any components of TEB localise at the DNA damage (DD) sites with some data suggesting that Thrap3 and Bclaf1a do not localise ( Beli *et al.*, 2012), and neither does Erh ( Kavanaugh *et al.*, 2015). In fact, Thrap3 is suggested to be excluded from double strand breaks ( Beli *et al.*, 2012). However, another study showed that Bclaf1 interacts and co-localises with H2AX after IR in damage foci ( Lee *et al.*, 2012). Bclaf1 has also been reported to form a complex with phosphorylated BRCA1, downstream of its function as *ATM* splicing regulator, promoting splicing of DDR proteins such as ATRIP, BACH1 and EXO1 ( Savage *et al.*, 2014). The diverse function of Bclaf1 can be partially explained by itself being a subject of splicing regulation, with one specific splicing isoform implicated in regulation of tumour growth ( Zhou *et al.*, 2014).

Crosslinks can not only be used to refine low or medium-resolution structures, but also aid the generation of protein models from their amino acid sequences ( Liu *et al.*, 2020; Orbán-Németh *et al.*, 2018). The crosslinking data presented here could be incorporated as C _α_-C _α_ distance restraints using I-TASSER ( Roy *et al.*, 2010; Yang *et al.*, 2015; Zhang, 2008) to refine the preliminary protein models we generated and improve their reliability. If satisfactory model structures are obtained, XL data can again be exploited through the HADDOCK platform for inter-molecular docking of the TEB complex subunits ( van Zundert *et al.*, 2016). However, due to lack of confident intramolecular XLs for Bclaf1 or available resolved structure of its homologues, its modelling may not be accurate enough to perform this task successfully.

In summary, we have performed XL-MS on endogenous protein complexes to derive useful topological information. We have shown that Thrap3, Bclaf1 and Erh interact directly with each other to form a tight protein assembly and we have identified their interaction interfaces though XL-MS. Abnormal splicing events are often observed in cancer cells and have been involved in many types of cancer. Hence, characterisation of the interactions between these proteins will be useful for better understanding their role in oncogenesis

## Methods

### Cell culture

Mouse embryonic stem cells (mESCs) were cultured by StemCell Technologies Inc. (Cambridge, UK).

### Immunoprecipitation and crosslinking

Protein G-Dynabeads (#10004D, Invitrogen) were prepared by coupling to antibodies against specific target protein in Table 2 for 15 minutes at room temperature (RT). Nuclear extraction was performed with isotonic buffer containing 10mM Tris-HCl, 10mM NaCl, 1.5mM MgCl2, 0.34M Sucrose, 1mM dithiothreitol (DTT), Halt Protease inhibitors (Thermo Scientific) and 0.05% NP-40. Nuclei were lysed for 10 minutes on ice with lysis buffer composed of 50 mM TrisHCl pH 8.0, 450 mM NaCl, 1 mM EDTA, 1 mM DTT, Halt Protease inhibitors (Thermo Scientific). NaCl was diluted to 150mM for immunoprecipitation (IP) for experiments 1-3 and 5, but kept at 450mM for experiment 4. Protein concentration was measured by Quick Start™ Bradford 1x Dye Reagent (#5000205, Biorad) following the manufactures guidelines. IP was performed from 10–60 mg protein depending on the experiment for 1–2 hours at 4°C. Crosslinking reagent disuccinimidyl sulfoxide (DSSO) (#A33545, ThermoFisher) was dissolved in DMSO at 50mM. The crosslinking was performed at 1mM (experiments 1-4) or 5mM (experiment 5) on the IP sample while still coupled to the beads, with DMSO final concertation being 2% at RT for 1 hour. The reaction was quenched with 125mM Tris-HCl pH 8.0 at RT for 15 minutes. The beads were washed three times with IPP150 (150mM NaCl, 10mM Tris-HCl, 0.1% NP40). For samples that were destined for MS analysis, the beads were washed and digested as previously described in Hillier *et al.* (2019) with a modified digestion schedule: Lys C digestion overnight, followed by Trypsin for approximately 4 hours, two times and a Trypsin overnight digestion.

**Table 2.** The antibodies used in immunoprecipitation (IP).

Target	Name	Catalogue number	RRID	Type	Species in which the antibody was raised	Company
**Arid1a**	PSG3	sc-32761	AB_673396	Monoclonal	Mouse	Santa Cruz Biotechnology
**N/A**	Mouse-IgG	12-371	AB_145840	Polyclonal	Mouse	Millipore

### Mass spectrometry

Peptides generated by trypsin digestion were fractionated with the Pierce High pH Reversed-Phase Peptide Fractionation Kit (#84868, ThermoFisher) according to manufacturer instructions, and eight fractions were collected and dried. Liquid chromatography mass spectrometry (LC-MS) analysis was performed on the Dionex UltiMate 3000 UHPLC system coupled with the Orbitrap Lumos Mass Spectrometer (Thermo Scientific). Each peptide fraction was reconstituted in 15 μL 0.1% formic acid and loaded to the Acclaim PepMap 100, 100 μm × 2 cm C18, 5 μm trapping column at 10 μL/min flow rate of 0.1% formic acid loading buffer. The sample was then subjected to a gradient elution on the EASY-Spray C18 capillary column (75 μm × 50 cm, 2 μm) at 50°C. Mobile phase A was 0.1% formic acid and mobile phase B was 80% acetonitrile, 0.1% formic acid. The gradient separation method at flow rate 300 nL/min was as follows: for 90 minutes gradient from 5%–38% B, for 10 minutes up to 95% B, for 5 minutes isocratic at 95% B, re-equilibration to 5% B in 5 minutes, for 10 minutes isocratic at 5% B. MS scans were acquired at a mass resolution of 120,000 and precursors between 375–1,600 m/z and charge equal or higher than +3 were isolated for collision-induced dissociation (CID) fragmentation with quadrupole isolation width 1.6 Th in the top speed mode in cycles of 5 seconds. Collision energy was set at 25%. Fragments with targeted mass difference of 31.9721 (DSSO crosslinker) were further subjected to CID fragmentation at the MS3 level with collision energy 35%, iontrap detection and MS2 isolation window 2 Th. Two precursor groups were selected with both ions in the pair. Targeted MS precursors were dynamically excluded for further isolation and activation for 30 seconds with 10 ppm mass tolerance.

### Mass spectrometry and crosslinking data analysis

MS raw data from mESCs IPs was analysed using Proteome Discoverer 2.4 (#OPTON-30945; Thermo Scientific). XLinkX was used to search crosslinked peptides pairs ( Liu *et al.*, 2015) for tryptic peptides with a minimum peptide length of 5 aa and maximum of 2 miss cleavages with dynamic modifications set to as oxidation of methionine (+15.995 Da) at 1% FDR. Precursor mass tolerance set to 20ppm, FTMS fragment mass tolerance to 30ppm, and ITMS fragment tolerance to 0.5 Da. Sequest was used for general MS2 search for tryptic peptides with a minimum peptide length of 6 aa and maximum of 2 missed cleavages with DSSO modifications followed by Target Decoy PSM Validator set at 1% FDR. Dynamic modifications were set as oxidation of methionine (+15.995 Da), DSSO hydrolysed for lysine (+176.014 Da) and DSSO quenched with Tris/dead end for lysine (+279.078 Da). The searches were performed against full UniProt Mouse database (August 2019) together with cRAP contaminant database. As an alternative to Proteome Discoverer 2.4 with XLinkX and Sequest, open-access software such as MaxQuant ( Cox & Mann, 2008) pLink ( Chen *et al*., 2019), XQuest/xProphet ( Leitner *et al.*, 2014; Rinner *et al.*, 2008; Walzthoeni *et al.*, 2012), StravoX ( Götze *et al.*, 2012), MeroX ( Götze *et al.*, 2015), Kojak ( Hoopmann *et al.*, 2015), XiSEARCH ( Mendes *et al.*, 2019) or MaxLinker ( Yugandhar *et al.*, 2020) could be used for XL identification.

The mass spectrometry proteomics data have been deposited to the ProteomeXchange Consortium via the PRIDE ( Perez-Riverol *et al.*, 2019) partner repository with the dataset identifier PXD027611 (see underlying data).

### Crosslinking mapping, protein modelling and visualisation

Annotations of domains and disorder regions of Thrap3, Bclaf1 and Erh as reported in Pfam database (v33.1) ( Mistry *et al.*, 2021). The illustration of the domains was made using DOG 2.0 ( Ren *et al.*, 2009). Two dimensional visualisation of crosslinks was performed using xiVIEW online tool ( Graham *et al.*, 2019). The crosslinks visualised were filtered so at least one of the proteins from the cross-linked pair is a member of the TEB complex and the site of the cross-linked pair was observed in more than one experiment. Structural models of Bclaf1 and Thrap3 were predicted by Robetta online tool with TrRefineRosetta modelling method for Bclaf1, and comparative modelling and *ab initio* modelling for Thrap3 ( Raman *et al.*, 2009; Song *et al.*, 2013). PDB files of the model and the available structures were visualised using UCSF Chimera package ( Pettersen *et al.*, 2004) and crosslinks were mapped using a Chimera plug-in Xlink Analyzer ( Kosinski *et al.*, 2015). Chimera was developed by the Resource for Biocomputing, Visualization, and Informatics at the University of California, San Francisco (supported by NIGMS P41-GM103311).

## Data Availability

The underlying data has been deposited in the ProteomeXchange Consortium via the PRIDE partner repository, accession number PXD027611: https://identifiers.org/pride.project:PXD027611.
