# Supplementary material for: Identifying and characterising Thrap3, Bclaf1 and Erh interactions using cross-linking mass spectrometry
Source: Wellcome Open Res. 2023 Jan 6;6:260. Originally published 2021 Oct 12. [Version 2] doi: 10.12688/wellcomeopenres.17160.2 (PMC9270653; doi:10.12688/wellcomeopenres.17160.2)
Supplement: Peer review history

# Peer review: Reviewer response for version 2

**DOI:** 10.21956/wellcomeopenres.20372.r53966

**Published:** 2023-01-11

**Authors:** Alexander Leitner

**Affiliations:** 1 Institute of Molecular Systems Biology, Department of Biology, ETH Zurich, Zurich, Switzerland

The changes to the text are appreciated. I have no further comments.

Is the work clearly and accurately presented and does it cite the current literature?

Yes

If applicable, is the statistical analysis and its interpretation appropriate?

Not applicable

Are all the source data underlying the results available to ensure full reproducibility?

Yes

Is the study design appropriate and is the work technically sound?

Yes

Are the conclusions drawn adequately supported by the results?

Yes

Are sufficient details of methods and analysis provided to allow replication by others?

Yes

Reviewer Expertise:

cross-linking mass spectrometry; proteomics; structural biology; protein-protein interactions, protein-RNA interactions

I confirm that I have read this submission and believe that I have an appropriate level of expertise to confirm that it is of an acceptable scientific standard.

# Peer review: Reviewer response for version 1

**DOI:** 10.21956/wellcomeopenres.18954.r51240

**Published:** 2022-07-08

**Authors:** J. Mark Skehel

**Affiliations:** 1 Francis Crick Institute, London, UK

The manuscript by Shcherbakova *et al.*, describes the use of chemical cross-linking to study the protein interactions of proteins and protein complexes at endogenous levels following affinity purification (IP-XL-MS).  The manuscript is well written and straightforward to follow. The study sets out to investigate the interactions of the BAF complex, by immunoprecipitating Arid1a, a scaffolding protein of the BAF complex.  Unfortunately, the experiment yielded too few cross-links of the BAF complex to enable detailed topological investigation, however, three proteins, Thrap3, Bclaf1, and Erh were identified with numerous cross-links, suggesting that they may form a previously unidentified complex.  In all, five experiments were carried out yielding a total of 121 cross-links.  Of these, 24 CSMs (cross-link spectral matches) were attributed to subunits of this putative complex.

 The study highlights an important aspect of structural mass spectrometry, namely that chemical cross-linking enables the structural analysis of intrinsically disordered proteins and disordered regions within a protein.  Thrap3 and Bclaf1 are predicted to have extensively disordered regions, which makes it very difficult if not impossible to study the structures by crystallography or cryo-electron microscopy.  While the data here provide limited intra and inter cross-links, they are starting point for more extensive investigation of this putative new complex.

 Raw data underlying this work has been deposited in the ProteomeXchange Consortium via the PRIDE repository.

 Minor comments:

  

1. The authors suggest naming the putative Bclaf1a-Erh-Thrap3 complex, the BET complex.  This is potentially confusing, as there are already the Bromodomain and Extra-Terminal domain (BET) proteins (BRD2, BRD3, BRD4, and BRDT).  Perhaps an alternative nomenclature would be better.
2. Table 1 should additionally show the confidence score attributed to a CSM by the software used for data analysis (XLinkX). The legend also refers to 4 experiments where five are shown.
3. The authors have employed the Robetta protein modelling tool to predict possible structures for these intrinsically disordered proteins.  It would be useful to compare these structures with those predicted by AlphaFold.
4. Searches were performed against the full UniProt Mouse database.  Potentially an alternative approach would have been to characterise the IP obtained using Arid1a and build a more targeted database against which to search the data.
5. The authors specify how protein concentrations were measured, but don’t say how much protein was used for the IP.  Additionally, a value of 1mM is given for the cross-linking reagent, how does this relate to a protein: cross-linker ratio?  Were several concentration ratios investigated?
6. On page 11, the authors mention that alternative software is available for data analysis.  This list is not extensive and should at least include other applications such as Xi, StavroX, etc.
7. The authors get slightly fixated on the distance restraint of 37Å.  This is a lenient value and may not have any particular importance for these disordered proteins.
8. For the cross-linking data analysis, it may prove useful to include serine and threonine in the reaction specificity for DSSO.

Is the work clearly and accurately presented and does it cite the current literature?

Yes

If applicable, is the statistical analysis and its interpretation appropriate?

Yes

Are all the source data underlying the results available to ensure full reproducibility?

Yes

Is the study design appropriate and is the work technically sound?

Yes

Are the conclusions drawn adequately supported by the results?

Yes

Are sufficient details of methods and analysis provided to allow replication by others?

Yes

Reviewer Expertise:

Proteomics, structural mass spectrometry

I confirm that I have read this submission and believe that I have an appropriate level of expertise to confirm that it is of an acceptable scientific standard.

# Response

**Published:** 2022-11-25

**Authors:** Liudmila Shcherbakova

**Affiliations:** The Institute of Cancer Research and The Royal Marsden NHS Foundation Trust, UK

- The authors suggest naming the putative Bclaf1a-Erh-Thrap3 complex, the BET complex.  This is potentially confusing, as there are already the Bromodomain and Extra-Terminal domain (BET) proteins (BRD2, BRD3, BRD4, and BRDT).  Perhaps an alternative nomenclature would be better.

*We thank the reviewer for pointing this out. We have now changed the name to TEB complex throughout the text*.

- Table 1 should additionally show the confidence score attributed to a CSM by the software used for data analysis (XLinkX). The legend also refers to 4 experiments where five are shown.

*The XlinkX Target Decoy PSM Validator was set at 1% FDR as reported in the methods. All crosslinks reported in table 1 fulfilled the 1% FDR cut-off. This has now been added to the legend for clarity and the number of experiments (5) has also been corrected.*

- The authors have employed the Robetta protein modelling tool to predict possible structures for these intrinsically disordered proteins. It would be useful to compare these structures with those predicted by AlphaFold.

*AlphaFold was released after the manuscript had been submitted, but we agree with the reviewer that it would be interesting to compare the modelled structures with the AlphaFold predicted structures. AlphaFold structures for human THRAP3 and BCLAF1 look more disordered than those presented in the paper for the mouse counterparts but retained a few alpha helixes in the central region of both THRAP3 and BCLAF1, similarly to the structures generated by Robetta. *

- Searches were performed against the full UniProt Mouse database.  Potentially an alternative approach would have been to characterise the IP obtained using Arid1a and build a more targeted database against which to search the data.

*Originally, since the experiments were designed around the BAF complex, we performed searches using a database restricted to BAF subunits and compared them with the full database search. The restricted database search did not result in a substantial improvement in crosslink assignment for BAF complex subunits and therefore the full mouse database was used to search for additional crosslinks.  *

- The authors specify how protein concentrations were measured, but don’t say how much protein was used for the IP.  Additionally, a value of 1mM is given for the cross-linking reagent, how does this relate to a protein: cross-linker ratio?  Were several concentration ratios investigated?

*We have now specified that 10-60 mg of nuclear lysate were used for the IP depending on the yield of the protein extraction on the day of the experiment.*

*Protein concentration was not measured after the IP as proteins were crosslinked and digested on the beads. Therefore, we are unable to provide an accurate protein to cross-linker ratio. We also tried 5 mM DSSO concentration (for experiment 5, now clarified in methods). An increase of ~1.8 times in crosslinks was observed. Although it is a substantial improvement, it was not proportional to the 5-fold increase in amount of crosslinker and was therefore deemed to not be cost-effective.*

- On page 11, the authors mention that alternative software is available for data analysis.  This list is not extensive and should at least include other applications such as Xi, StavroX, etc.

*We have now added other software for XL data analysis: ”As an alternative to Proteome Discoverer 2.4 with XLinkX and Sequest, open-access software such as MaxQuant (Cox and Mann, 2008), pLink (Chen et al., 2019), XQuest/xProphet (Rinner et al., 2008, Walzthoeni et al., 2012, Leitner et al., 2014), StravoX (Götze et al., 2012), MeroX (Götze et al., 2015), Kojak (Hoopmann et al., 2015), XiSEARCH (Mendes et al., 2019) or MaxLinker (Yugandhar et al., 2020) could be used for XL identification.”*

- The authors get slightly fixated on the distance restraint of 37Å.  This is a lenient value and may not have any particular importance for these disordered proteins.

*We agree that 37 Å is a lax value. We have now relaxed the use of the 37 Å threshold throughout the manuscript. Further examination of our results has revealed that most of Thrap3 XLs satisfy a distance constraint of 32 Å is all models, and we have modified the manuscript accordingly.*

- For the cross-linking data analysis, it may prove useful to include serine and threonine in the reaction specificity for DSSO.

*We thank the reviewer for this suggestion. We did not check for serine and threonine sites as we generally see less than 1% reactivity with these residues when using other reagents with similar reaction chemistry to DSSO such as TMT.*

References:

 CHEN, Z.-L., MENG, J.-M., CAO, Y., YIN, J.-L., FANG, R.-Q., FAN, S.-B., LIU, C., ZENG, W.-F., DING, Y.-H., TAN, D., WU, L., ZHOU, W.-J., CHI, H., SUN, R.-X., DONG, M.-Q. & HE, S.-M. 2019. A high-speed search engine pLink 2 with systematic evaluation for proteome-scale identification of cross-linked peptides. Nature Communications, 10, 3404.

 COX, J. & MANN, M. 2008. MaxQuant enables high peptide identification rates, individualized p.p.b.-range mass accuracies and proteome-wide protein quantification. Nature Biotechnology, 26, 1367-1372.

 GÖTZE, M., PETTELKAU, J., FRITZSCHE, R., IHLING, C. H., SCHÄFER, M. & SINZ, A. 2015. Automated Assignment of MS/MS Cleavable Cross-Links in Protein 3D-Structure Analysis. Journal of the American Society for Mass Spectrometry, 26, 83-97.

 GÖTZE, M., PETTELKAU, J., SCHAKS, S., BOSSE, K., IHLING, C. H., KRAUTH, F., FRITZSCHE, R., KÜHN, U. & SINZ, A. 2012. StavroX--a software for analyzing crosslinked products in protein interaction studies. J Am Soc Mass Spectrom, 23, 76-87.

 HOOPMANN, M. R., ZELTER, A., JOHNSON, R. S., RIFFLE, M., MACCOSS, M. J., DAVIS, T. N. & MORITZ, R. L. 2015. Kojak: Efficient Analysis of Chemically Cross-Linked Protein Complexes. Journal of Proteome Research, 14, 2190-2198.

 LEITNER, A., WALZTHOENI, T. & AEBERSOLD, R. 2014. Lysine-specific chemical cross-linking of protein complexes and identification of cross-linking sites using LC-MS/MS and the xQuest/xProphet software pipeline. Nat Protoc, 9, 120-37.

 MENDES, M. L., FISCHER, L., CHEN, Z. A., BARBON, M., O'REILLY, F. J., GIESE, S. H., BOHLKE-SCHNEIDER, M., BELSOM, A., DAU, T., COMBE, C. W., GRAHAM, M., EISELE, M. R., BAUMEISTER, W., SPECK, C. & RAPPSILBER, J. 2019. An integrated workflow for crosslinking mass spectrometry. Mol Syst Biol, 15, e8994.

 RINNER, O., SEEBACHER, J., WALZTHOENI, T., MUELLER, L. N., BECK, M., SCHMIDT, A., MUELLER, M. & AEBERSOLD, R. 2008. Identification of cross-linked peptides from large sequence databases. Nat Methods, 5, 315-8.

 WALZTHOENI, T., CLAASSEN, M., LEITNER, A., HERZOG, F., BOHN, S., FÖRSTER, F., BECK, M. & AEBERSOLD, R. 2012. False discovery rate estimation for cross-linked peptides identified by mass spectrometry. Nature Methods, 9, 901-903.

 YUGANDHAR, K., WANG, T.-Y., LEUNG, A. K.-Y., LANZ, M. C., MOTORYKIN, I., LIANG, J., SHAYHIDIN, E. E., SMOLKA, M. B., ZHANG, S. & YU, H. 2020. MaXLinker: Proteome-wide Cross-link Identifications with High Specificity and Sensitivity*. Molecular & Cellular Proteomics, 19, 554-568.

# Peer review: Reviewer response for version 1

**DOI:** 10.21956/wellcomeopenres.18954.r50940

**Published:** 2022-06-27

**Authors:** Alexander Leitner

**Affiliations:** 1 Institute of Molecular Systems Biology, Department of Biology, ETH Zurich, Zurich, Switzerland

In this contribution, Shcherbakova *et al. *report the characterization of the ternary interaction between mouse Thrap3, Bclaf1, and Erh by chemical cross-linking (XL) and mass spectrometry. The cross-linking data was obtained from an experiment that initially targeted the BAF complex, which did not yield sufficient cross-links for a detailed structural interpretation. Thrap3, Bclaf1, and Erh, however, were found to be connected through a mutually supporting set of cross-links, suggesting that the observed connections specify the binding interfaces in a ternary complex. The manuscript describes data generation from the IP-XL-MS experiment, for which results from multiple, independent experiments were combined. In total, 24 non-redundant peptide pairs involving at least one of the three proteins were identified repeatedly.

 Thrap3 and Bclaf1 are predicted to have large unstructured/intrinsically disordered regions, which makes it difficult to study their interactions by established structural biology methods. The XL data presented here serve as a starting point for further experiments, including integrative modeling approaches to define the organization of the complex. The role of Thrap3 and Bclaf1 in some splicing events may make the complex relevant for diseases such as cancer.

 The manuscript is clearly written and experiments are described in sufficient detail to enable replication. XL-MS results have been deposited to the PRIDE repository, facilitating reanalysis or reuse of the data.

 Minor comments that could be addressed in a revised version:

- On page 7, the authors discuss the potential ambiguity of XL data when it comes to site pairs on the same protein. While this is true, the case described here - a cross-link that connects partially overlapping sequences - needs to be inter-molecular (or a false positive identification). I would emphasize this more in the text.
- Several times throughout the manuscript, the authors mention that they used an upper distance of 37 Å to assess "compatibility" of a cross-link with available PDB structures or structure predictions, citing Liu et al., 2020, as a reference. I would like to point out that 37 Å is a quite lenient threshold, and upper distances of 30-32 Å are much more commonly used. Nevertheless, an interaction between largely unstructured proteins may sample a larger conformational space. Somewhat related to this, the authors discuss that intra-molecular links on Thrap3 were compatible with one of the proposed models (page 7). Since more than one model was predicted, how did the links fit to other models?
- In the methods section (page 10), an "IPP150" buffer is mentioned that needs to be defined. On the same page, "precursor selection ... with a mass resolution of 120,000" should be rephrased. Precursors were detected at a resolution of 120,000 but their selection for fragmentation is only dependent on the isolation width. "miss cleavages" should read "missed cleavages".
- The legend in Table 1 mentions four experiments, although the results from five are displayed.

Is the work clearly and accurately presented and does it cite the current literature?

Yes

If applicable, is the statistical analysis and its interpretation appropriate?

Not applicable

Are all the source data underlying the results available to ensure full reproducibility?

Yes

Is the study design appropriate and is the work technically sound?

Yes

Are the conclusions drawn adequately supported by the results?

Yes

Are sufficient details of methods and analysis provided to allow replication by others?

Yes

Reviewer Expertise:

cross-linking mass spectrometry; proteomics; structural biology; protein-protein interactions, protein-RNA interactions

I confirm that I have read this submission and believe that I have an appropriate level of expertise to confirm that it is of an acceptable scientific standard.

# Response

**Published:** 2022-11-25

**Authors:** Liudmila Shcherbakova

**Affiliations:** The Institute of Cancer Research and The Royal Marsden NHS Foundation Trust, UK

- On page 7, the authors discuss the potential ambiguity of XL data when it comes to site pairs on the same protein. While this is true, the case described here - a cross-link that connects partially overlapping sequences - needs to be inter-molecular (or a false positive identification). I would emphasize this more in the text.

*We have now emphasised that the cross-link that connects partially overlapping sequences corresponds to an inter-molecular connection (or a false positive identification).*

- Several times throughout the manuscript, the authors mention that they used an upper distance of 37 Å to assess "compatibility" of a cross-link with available PDB structures or structure predictions, citing Liu et al., 2020, as a reference. I would like to point out that 37 Å is a quite lenient threshold, and upper distances of 30-32 Å are much more commonly used. Nevertheless, an interaction between largely unstructured proteins may sample a larger conformational space. Somewhat related to this, the authors discuss that intra-molecular links on Thrap3 were compatible with one of the proposed models (page 7). Since more than one model was predicted, how did the links fit to other models?

*We thank the reviewer for his expert comment on the cross-link distance upper threshold.*

*The cross-links fit to the other 4 models was similar, with models 2,3 and 5 satisfying all 5/5 crosslinks at a distance of 32 Å and models 1 and 4 satisfying 4/5 crosslinks at 32 Å. We have now modified the text to include this information.*

- In the methods section (page 10), an "IPP150" buffer is mentioned that needs to be defined. On the same page, "precursor selection ... with a mass resolution of 120,000" should be rephrased. Precursors were detected at a resolution of 120,000 but their selection for fragmentation is only dependent on the isolation width. "miss cleavages" should read "missed cleavages".

*The IPP150 buffer composition* *(150 NaCl, 10mM Tris-HCl, 0.1% NP40)* *is now defined in the text.*

*We have rephrased the MS mass resolution and precursor selection parameters to* *: “MS scans were acquired at a mass resolution of 120,000 and precursors between 375-1,600 m/z and charge equal or higher than +3 were isolated for collision-induced dissociation (CID) fragmentation with quadrupole isolation width 1.6 Th in the top speed mode in cycles of 5 seconds.”*

*We have also replaced “miss cleavages” with “missed cleavages”.*

- The legend in Table 1 mentions four experiments, although the results from five are displayed

*The legend has been corrected.*
